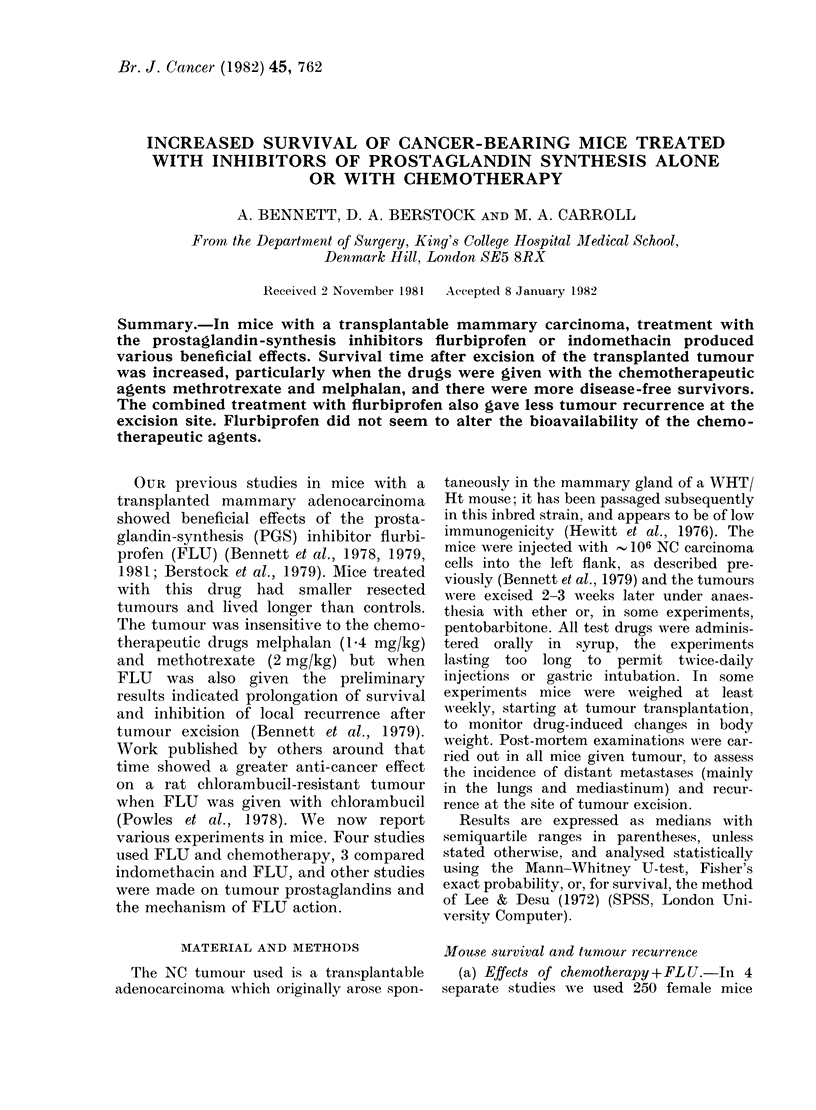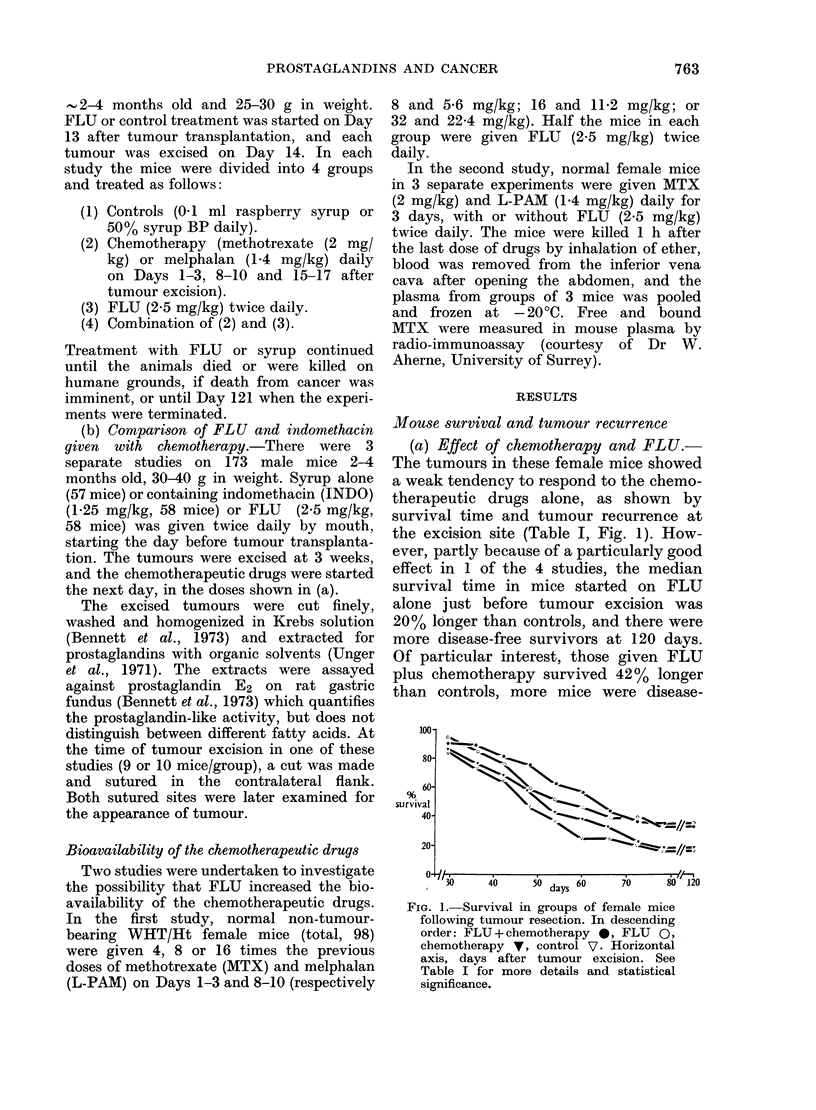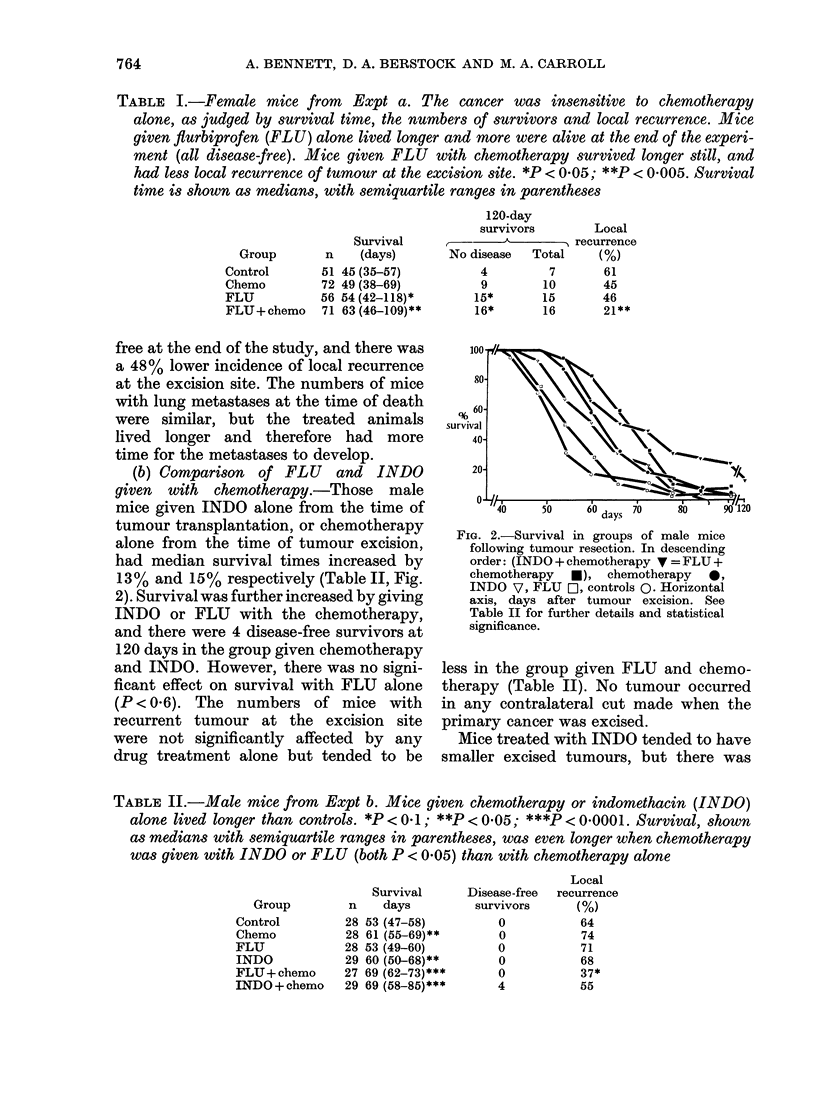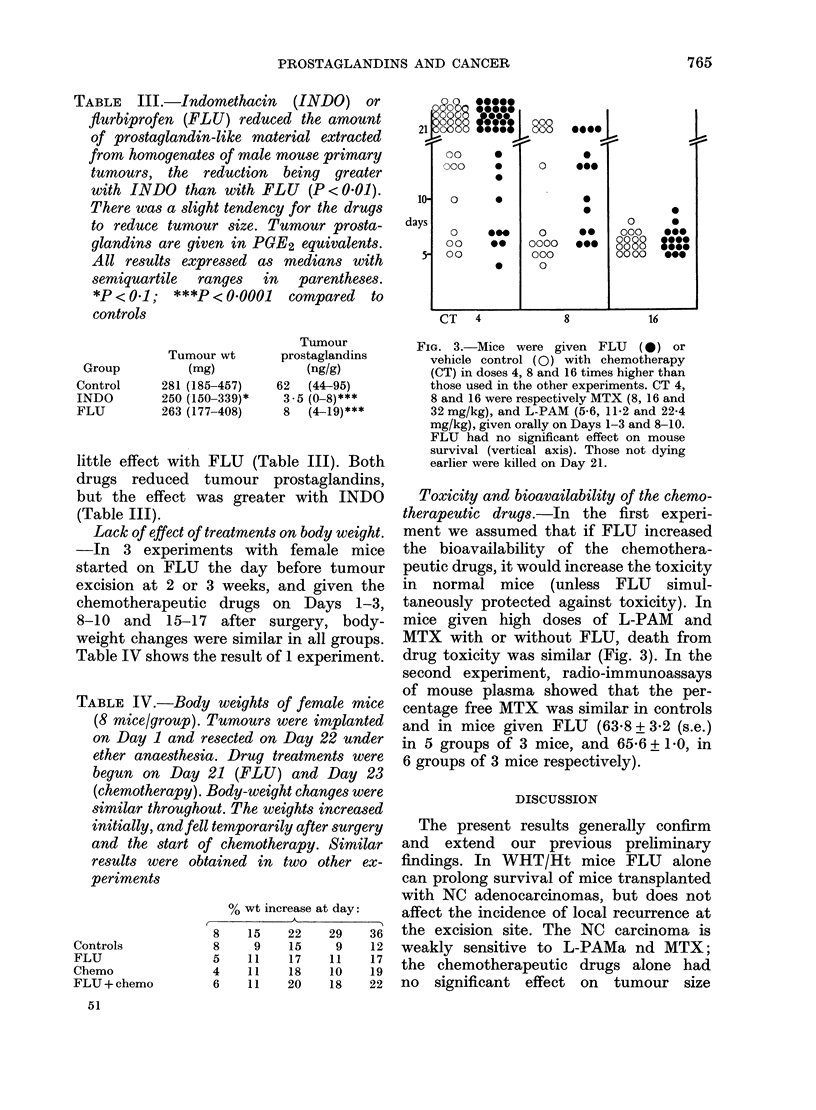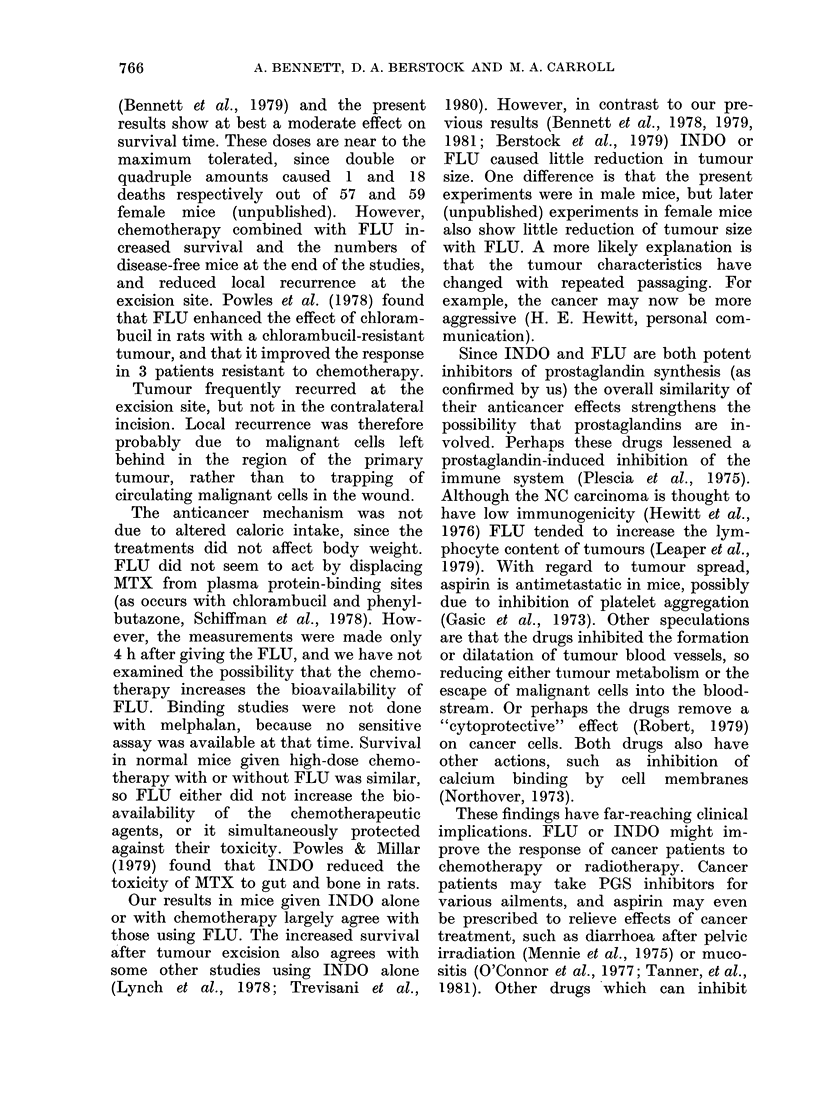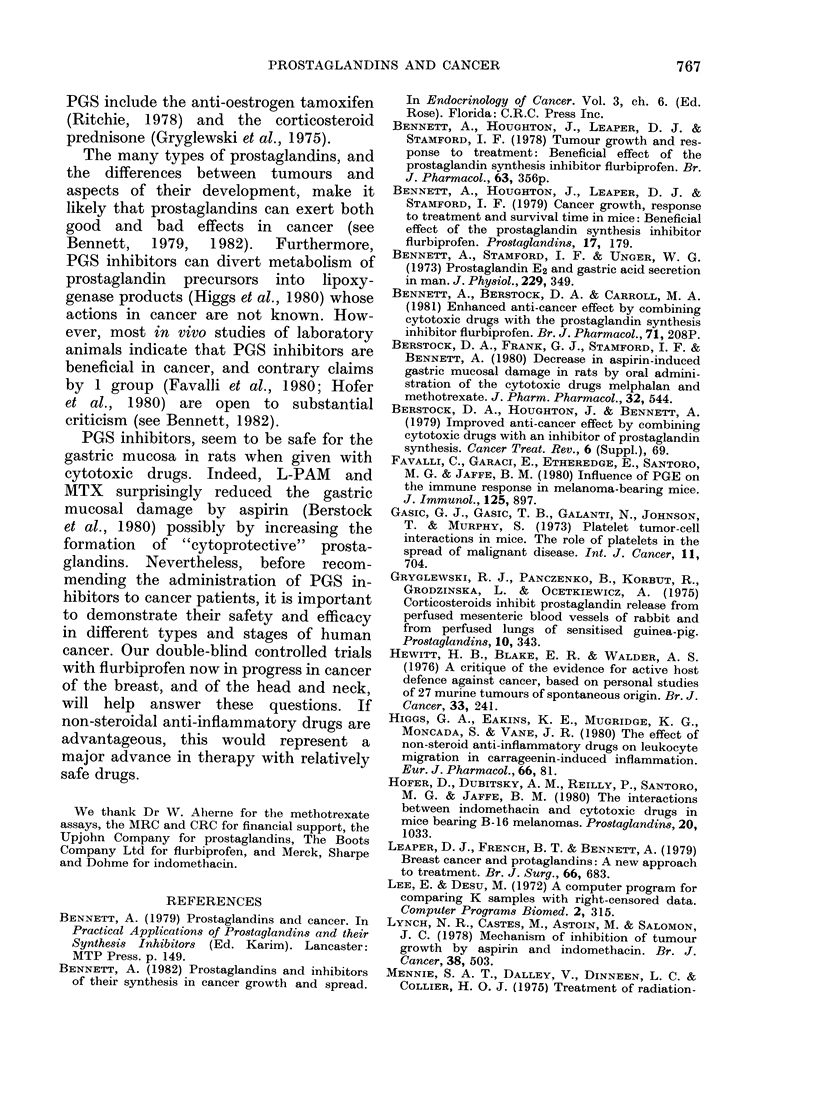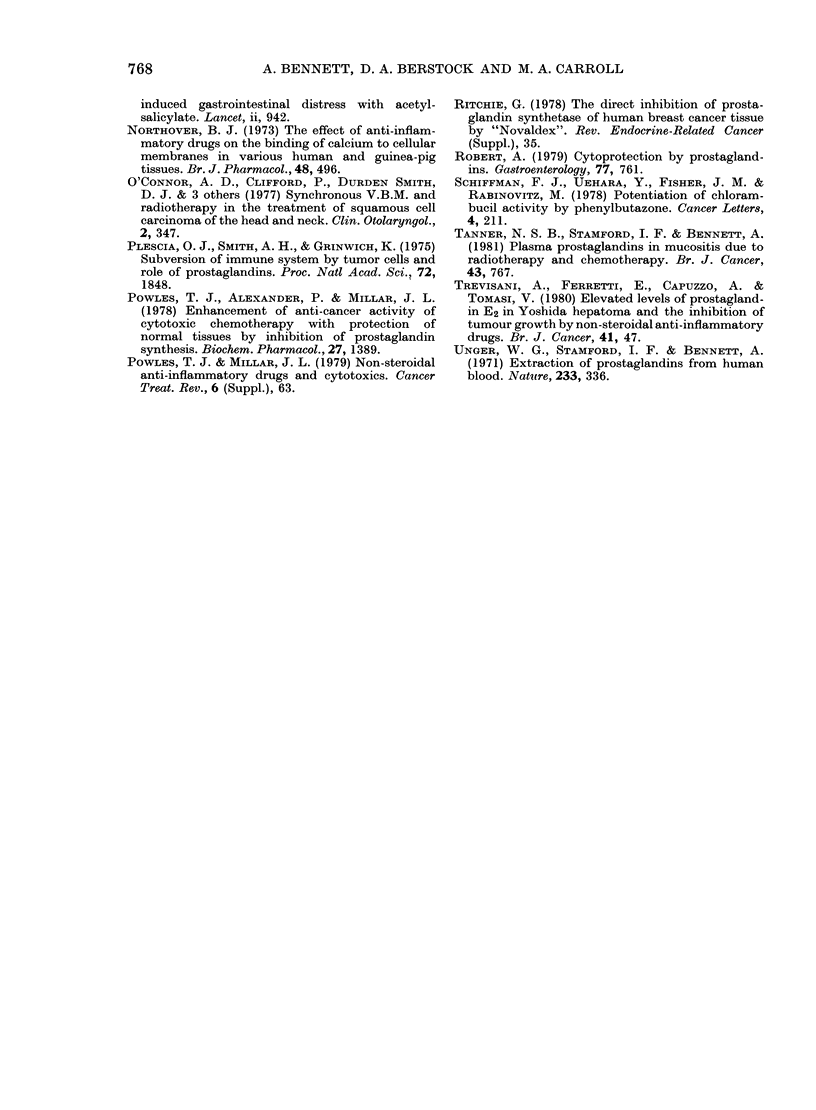# Increased survival of cancer-bearing mice treated with inhibitors of prostaglandin synthesis alone or with chemotherapy.

**DOI:** 10.1038/bjc.1982.118

**Published:** 1982-05

**Authors:** A. Bennett, D. A. Berstock, M. A. Carroll

## Abstract

In mice with a transplantable mammary carcinoma, treatment with the prostaglandin-synthesis inhibitors flurbiprofen or indomethacin produced various beneficial effects. Survival time after excision of the transplanted tumour was increase, particularly when the drugs were given with the chemotherapeutic agents methrotrexate and melphalan, and there were more disease-free survivors. The combined treatment with flurbiprofen also gave less tumour recurrence at the excision site. Flurbiprofen did not seem to alter the bioavailability of the chemotherapeutic agents.


Br. J. Cancer (1982) 45, 762

### INCREASED SURVIVAL OF CANCER-BEARING MICE TREATED WITH INHIBITORS OF PROSTAGLANDIN SYNTHESIS ALONE OR WITH CHEMOTHERAPY

A. BENNETT, D. A. BERSTOCK AND M. A. CARROLL

*From the Department of Surgery, King's College Hospital Medical School, Denmark Hill, London SE5 8RX*

Received 2 November 1981 Accepted 8 January 1982

**Summary.—In mice with a transplantable mammary carcinoma, treatment with the prostaglandin-synthesis inhibitors flurbiprofen or indomethacin produced various beneficial effects. Survival time after excision of the transplanted tumour was increased, particularly when the drugs were given with the chemotherapeutic agents methrotrexate and melphalan, and there were more disease-free survivors. The combined treatment with flurbiprofen also gave less tumour recurrence at the excision site. Flurbiprofen did not seem to alter the bioavailability of the chemotherapeutic agents.**

OUR previous studies in mice with a transplanted mammary adenocarcinoma showed beneficial effects of the prostaglandin-synthesis (PGS) inhibitor flurbiprofen (FLU) (Bennett *et al.*, 1978, 1979, 1981; Berstock *et al.*, 1979). Mice treated with this drug had smaller resected tumours and lived longer than controls. The tumour was insensitive to the chemotherapeutic drugs melphalan (1·4 mg/kg) and methotrexate (2 mg/kg) but when FLU was also given the preliminary results indicated prolongation of survival and inhibition of local recurrence after tumour excision (Bennett *et al.*, 1979). Work published by others around that time showed a greater anti-cancer effect on a rat chlorambucil-resistant tumour when FLU was given with chlorambucil (Powles *et al.*, 1978). We now report various experiments in mice. Four studies used FLU and chemotherapy, 3 compared indomethacin and FLU, and other studies were made on tumour prostaglandins and the mechanism of FLU action.

### MATERIAL AND METHODS

The NC tumour used is a transplantable adenocarcinoma which originally arose spontaneously in the mammary gland of a WHT/Ht mouse; it has been passaged subsequently in this inbred strain, and appears to be of low immunogenicity (Hewitt *et al.*, 1976). The mice were injected with $\sim 10^6$ NC carcinoma cells into the left flank, as described previously (Bennett *et al.*, 1979) and the tumours were excised 2–3 weeks later under anaesthesia with ether or, in some experiments, pentobarbitone. All test drugs were administered orally in syrup, the experiments lasting too long to permit twice-daily injections or gastric intubation. In some experiments mice were weighed at least weekly, starting at tumour transplantation, to monitor drug-induced changes in body weight. Post-mortem examinations were carried out in all mice given tumour, to assess the incidence of distant metastases (mainly in the lungs and mediastinum) and recurrence at the site of tumour excision.

Results are expressed as medians with semiquartile ranges in parentheses, unless stated otherwise, and analysed statistically using the Mann–Whitney U-test, Fisher's exact probability, or, for survival, the method of Lee & Desu (1972) (SPSS, London University Computer).

### *Mouse survival and tumour recurrence*

(a) *Effects of chemotherapy + FLU.*—In 4 separate studies we used 250 female mice

~2–4 months old and 25–30 g in weight. FLU or control treatment was started on Day 13 after tumour transplantation, and each tumour was excised on Day 14. In each study the mice were divided into 4 groups and treated as follows:

(1) Controls (0·1 ml raspberry syrup or 50% syrup BP daily).
(2) Chemotherapy (methotrexate (2 mg/kg) or melphalan (1·4 mg/kg) daily on Days 1–3, 8–10 and 15–17 after tumour excision).
(3) FLU (2·5 mg/kg) twice daily.
(4) Combination of (2) and (3).

Treatment with FLU or syrup continued until the animals died or were killed on humane grounds, if death from cancer was imminent, or until Day 121 when the experiments were terminated.

(b) *Comparison of FLU and indomethacin given with chemotherapy.*—There were 3 separate studies on 173 male mice 2–4 months old, 30–40 g in weight. Syrup alone (57 mice) or containing indomethacin (INDO) (1·25 mg/kg, 58 mice) or FLU (2·5 mg/kg, 58 mice) was given twice daily by mouth, starting the day before tumour transplantation. The tumours were excised at 3 weeks, and the chemotherapeutic drugs were started the next day, in the doses shown in (a).

The excised tumours were cut finely, washed and homogenized in Krebs solution (Bennett *et al.*, 1973) and extracted for prostaglandins with organic solvents (Unger *et al.*, 1971). The extracts were assayed against prostaglandin $E_2$ on rat gastric fundus (Bennett *et al.*, 1973) which quantifies the prostaglandin-like activity, but does not distinguish between different fatty acids. At the time of tumour excision in one of these studies (9 or 10 mice/group), a cut was made and sutured in the contralateral flank. Both sutured sites were later examined for the appearance of tumour.

### *Bioavailability of the chemotherapeutic drugs*

Two studies were undertaken to investigate the possibility that FLU increased the bioavailability of the chemotherapeutic drugs. In the first study, normal non-tumour-bearing WHT/Ht female mice (total, 98) were given 4, 8 or 16 times the previous doses of methotrexate (MTX) and melphalan (L-PAM) on Days 1–3 and 8–10 (respectively 8 and 5·6 mg/kg; 16 and 11·2 mg/kg; or 32 and 22·4 mg/kg). Half the mice in each group were given FLU (2·5 mg/kg) twice daily.

In the second study, normal female mice in 3 separate experiments were given MTX (2 mg/kg) and L-PAM (1·4 mg/kg) daily for 3 days, with or without FLU (2·5 mg/kg) twice daily. The mice were killed 1 h after the last dose of drugs by inhalation of ether, blood was removed from the inferior vena cava after opening the abdomen, and the plasma from groups of 3 mice was pooled and frozen at −20°C. Free and bound MTX were measured in mouse plasma by radio-immunoassay (courtesy of Dr W. Aherne, University of Surrey).

### RESULTS

### *Mouse survival and tumour recurrence*

(*a*) *Effect of chemotherapy and FLU.*—The tumours in these female mice showed a weak tendency to respond to the chemotherapeutic drugs alone, as shown by survival time and tumour recurrence at the excision site (Table I, Fig. 1). However, partly because of a particularly good effect in 1 of the 4 studies, the median survival time in mice started on FLU alone just before tumour excision was 20% longer than controls, and there were more disease-free survivors at 120 days. Of particular interest, those given FLU plus chemotherapy survived 42% longer than controls, more mice were disease-

FIG. 1.—Survival in groups of female mice following tumour resection. In descending order: FLU + chemotherapy ●, FLU ○, chemotherapy ▼, control ▽. Horizontal axis, days after tumour excision. See Table I for more details and statistical significance.

TABLE I.—*Female mice from Expt a. The cancer was insensitive to chemotherapy alone, as judged by survival time, the numbers of survivors and local recurrence. Mice given flurbiprofen (FLU) alone lived longer and more were alive at the end of the experiment (all disease-free). Mice given FLU with chemotherapy survived longer still, and had less local recurrence of tumour at the excision site.* *$P < 0{\cdot}05$; **$P < 0{\cdot}005$. *Survival time is shown as medians, with semiquartile ranges in parentheses*

| Group | n | Survival (days) | 120-day survivors: No disease | 120-day survivors: Total | Local recurrence (%) |
|---|---|---|---|---|---|
| Control | 51 | 45 (35–57) | 4 | 7 | 61 |
| Chemo | 72 | 49 (38–69) | 9 | 10 | 45 |
| FLU | 56 | 54 (42–118)* | 15* | 15 | 46 |
| FLU+chemo | 71 | 63 (46–109)** | 16* | 16 | 21** |

free at the end of the study, and there was a 48% lower incidence of local recurrence at the excision site. The numbers of mice with lung metastases at the time of death were similar, but the treated animals lived longer and therefore had more time for the metastases to develop.

(*b*) *Comparison of FLU and INDO given with chemotherapy.*—Those male mice given INDO alone from the time of tumour transplantation, or chemotherapy alone from the time of tumour excision, had median survival times increased by 13% and 15% respectively (Table II, Fig. 2). Survival was further increased by giving INDO or FLU with the chemotherapy, and there were 4 disease-free survivors at 120 days in the group given chemotherapy and INDO. However, there was no significant effect on survival with FLU alone ($P < 0{\cdot}6$). The numbers of mice with recurrent tumour at the excision site were not significantly affected by any drug treatment alone but tended to be less in the group given FLU and chemotherapy (Table II). No tumour occurred in any contralateral cut made when the primary cancer was excised.

FIG. 2.—Survival in groups of male mice following tumour resection. In descending order: (INDO+chemotherapy ▼=FLU+chemotherapy ■), chemotherapy ●, INDO ▽, FLU □, controls ○. Horizontal axis, days after tumour excision. See Table II for further details and statistical significance.

Mice treated with INDO tended to have smaller excised tumours, but there was

TABLE II.—*Male mice from Expt b. Mice given chemotherapy or indomethacin (INDO) alone lived longer than controls.* *$P < 0{\cdot}1$; **$P < 0{\cdot}05$; ***$P < 0{\cdot}0001$. *Survival, shown as medians with semiquartile ranges in parentheses, was even longer when chemotherapy was given with INDO or FLU (both $P < 0{\cdot}05$) than with chemotherapy alone*

| Group | n | Survival days | Disease-free survivors | Local recurrence (%) |
|---|---|---|---|---|
| Control | 28 | 53 (47–58) | 0 | 64 |
| Chemo | 28 | 61 (55–69)** | 0 | 74 |
| FLU | 28 | 53 (49–60) | 0 | 71 |
| INDO | 29 | 60 (50–68)** | 0 | 68 |
| FLU+chemo | 27 | 69 (62–73)*** | 0 | 37* |
| INDO+chemo | 29 | 69 (58–85)*** | 4 | 55 |

TABLE III.—*Indomethacin (INDO) or flurbiprofen (FLU) reduced the amount of prostaglandin-like material extracted from homogenates of male mouse primary tumours, the reduction being greater with INDO than with FLU ($P < 0.01$). There was a slight tendency for the drugs to reduce tumour size. Tumour prostaglandins are given in $PGE_2$ equivalents. All results expressed as medians with semiquartile ranges in parentheses. *$P < 0.1$; ***$P < 0.0001$ compared to controls*

| Group | Tumour wt (mg) | Tumour prostaglandins (ng/g) |
|---|---|---|
| Control | 281 (185–457) | 62 (44–95) |
| INDO | 250 (150–339)* | 3·5 (0–8)*** |
| FLU | 263 (177–408) | 8 (4–19)*** |

little effect with FLU (Table III). Both drugs reduced tumour prostaglandins, but the effect was greater with INDO (Table III).

*Lack of effect of treatments on body weight.*—In 3 experiments with female mice started on FLU the day before tumour excision at 2 or 3 weeks, and given the chemotherapeutic drugs on Days 1–3, 8–10 and 15–17 after surgery, body-weight changes were similar in all groups. Table IV shows the result of 1 experiment.

TABLE IV.—*Body weights of female mice (8 mice/group). Tumours were implanted on Day 1 and resected on Day 22 under ether anaesthesia. Drug treatments were begun on Day 21 (FLU) and Day 23 (chemotherapy). Body-weight changes were similar throughout. The weights increased initially, and fell temporarily after surgery and the start of chemotherapy. Similar results were obtained in two other experiments*

| | % wt increase at day: | | | | |
|---|---|---|---|---|---|
| | 8 | 15 | 22 | 29 | 36 |
| Controls | 8 | 9 | 15 | 9 | 12 |
| FLU | 5 | 11 | 17 | 11 | 17 |
| Chemo | 4 | 11 | 18 | 10 | 19 |
| FLU+chemo | 6 | 11 | 20 | 18 | 22 |

FIG. 3.—Mice were given FLU (●) or vehicle control (○) with chemotherapy (CT) in doses 4, 8 and 16 times higher than those used in the other experiments. CT 4, 8 and 16 were respectively MTX (8, 16 and 32 mg/kg), and L-PAM (5·6, 11·2 and 22·4 mg/kg), given orally on Days 1–3 and 8–10. FLU had no significant effect on mouse survival (vertical axis). Those not dying earlier were killed on Day 21.

*Toxicity and bioavailability of the chemotherapeutic drugs.*—In the first experiment we assumed that if FLU increased the bioavailability of the chemotherapeutic drugs, it would increase the toxicity in normal mice (unless FLU simultaneously protected against toxicity). In mice given high doses of L-PAM and MTX with or without FLU, death from drug toxicity was similar (Fig. 3). In the second experiment, radio-immunoassays of mouse plasma showed that the percentage free MTX was similar in controls and in mice given FLU ($63·8 \pm 3·2$ (s.e.) in 5 groups of 3 mice, and $65·6 \pm 1·0$, in 6 groups of 3 mice respectively).

### DISCUSSION

The present results generally confirm and extend our previous preliminary findings. In WHT/Ht mice FLU alone can prolong survival of mice transplanted with NC adenocarcinomas, but does not affect the incidence of local recurrence at the excision site. The NC carcinoma is weakly sensitive to L-PAMa nd MTX; the chemotherapeutic drugs alone had no significant effect on tumour size

(Bennett *et al.*, 1979) and the present results show at best a moderate effect on survival time. These doses are near to the maximum tolerated, since double or quadruple amounts caused 1 and 18 deaths respectively out of 57 and 59 female mice (unpublished). However, chemotherapy combined with FLU increased survival and the numbers of disease-free mice at the end of the studies, and reduced local recurrence at the excision site. Powles *et al.* (1978) found that FLU enhanced the effect of chlorambucil in rats with a chlorambucil-resistant tumour, and that it improved the response in 3 patients resistant to chemotherapy.

Tumour frequently recurred at the excision site, but not in the contralateral incision. Local recurrence was therefore probably due to malignant cells left behind in the region of the primary tumour, rather than to trapping of circulating malignant cells in the wound.

The anticancer mechanism was not due to altered caloric intake, since the treatments did not affect body weight. FLU did not seem to act by displacing MTX from plasma protein-binding sites (as occurs with chlorambucil and phenylbutazone, Schiffman *et al.*, 1978). However, the measurements were made only 4 h after giving the FLU, and we have not examined the possibility that the chemotherapy increases the bioavailability of FLU. Binding studies were not done with melphalan, because no sensitive assay was available at that time. Survival in normal mice given high-dose chemotherapy with or without FLU was similar, so FLU either did not increase the bioavailability of the chemotherapeutic agents, or it simultaneously protected against their toxicity. Powles & Millar (1979) found that INDO reduced the toxicity of MTX to gut and bone in rats.

Our results in mice given INDO alone or with chemotherapy largely agree with those using FLU. The increased survival after tumour excision also agrees with some other studies using INDO alone (Lynch *et al.*, 1978; Trevisani *et al.*, 1980). However, in contrast to our previous results (Bennett *et al.*, 1978, 1979, 1981; Berstock *et al.*, 1979) INDO or FLU caused little reduction in tumour size. One difference is that the present experiments were in male mice, but later (unpublished) experiments in female mice also show little reduction of tumour size with FLU. A more likely explanation is that the tumour characteristics have changed with repeated passaging. For example, the cancer may now be more aggressive (H. E. Hewitt, personal communication).

Since INDO and FLU are both potent inhibitors of prostaglandin synthesis (as confirmed by us) the overall similarity of their anticancer effects strengthens the possibility that prostaglandins are involved. Perhaps these drugs lessened a prostaglandin-induced inhibition of the immune system (Plescia *et al.*, 1975). Although the NC carcinoma is thought to have low immunogenicity (Hewitt *et al.*, 1976) FLU tended to increase the lymphocyte content of tumours (Leaper *et al.*, 1979). With regard to tumour spread, aspirin is antimetastatic in mice, possibly due to inhibition of platelet aggregation (Gasic *et al.*, 1973). Other speculations are that the drugs inhibited the formation or dilatation of tumour blood vessels, so reducing either tumour metabolism or the escape of malignant cells into the bloodstream. Or perhaps the drugs remove a "cytoprotective" effect (Robert, 1979) on cancer cells. Both drugs also have other actions, such as inhibition of calcium binding by cell membranes (Northover, 1973).

These findings have far-reaching clinical implications. FLU or INDO might improve the response of cancer patients to chemotherapy or radiotherapy. Cancer patients may take PGS inhibitors for various ailments, and aspirin may even be prescribed to relieve effects of cancer treatment, such as diarrhoea after pelvic irradiation (Mennie *et al.*, 1975) or mucositis (O'Connor *et al.*, 1977; Tanner, *et al.*, 1981). Other drugs which can inhibit

PGS include the anti-oestrogen tamoxifen (Ritchie, 1978) and the corticosteroid prednisone (Gryglewski *et al.*, 1975).

The many types of prostaglandins, and the differences between tumours and aspects of their development, make it likely that prostaglandins can exert both good and bad effects in cancer (see Bennett, 1979, 1982). Furthermore, PGS inhibitors can divert metabolism of prostaglandin precursors into lipoxygenase products (Higgs *et al.*, 1980) whose actions in cancer are not known. However, most *in vivo* studies of laboratory animals indicate that PGS inhibitors are beneficial in cancer, and contrary claims by 1 group (Favalli *et al.*, 1980; Hofer *et al.*, 1980) are open to substantial criticism (see Bennett, 1982).

PGS inhibitors, seem to be safe for the gastric mucosa in rats when given with cytotoxic drugs. Indeed, L-PAM and MTX surprisingly reduced the gastric mucosal damage by aspirin (Berstock *et al.*, 1980) possibly by increasing the formation of "cytoprotective" prostaglandins. Nevertheless, before recommending the administration of PGS inhibitors to cancer patients, it is important to demonstrate their safety and efficacy in different types and stages of human cancer. Our double-blind controlled trials with flurbiprofen now in progress in cancer of the breast, and of the head and neck, will help answer these questions. If non-steroidal anti-inflammatory drugs are advantageous, this would represent a major advance in therapy with relatively safe drugs.

We thank Dr W. Aherne for the methotrexate assays, the MRC and CRC for financial support, the Upjohn Company for prostaglandins, The Boots Company Ltd for flurbiprofen, and Merck, Sharpe and Dohme for indomethacin.

### REFERENCES

BENNETT, A. (1979) Prostaglandins and cancer. In *Practical Applications of Prostaglandins and their Synthesis Inhibitors* (Ed. Karim). Lancaster: MTP Press. p. 149.

BENNETT, A. (1982) Prostaglandins and inhibitors of their synthesis in cancer growth and spread. In *Endocrinology of Cancer*. Vol. 3, ch. 6. (Ed. Rose). Florida: C.R.C. Press Inc.

BENNETT, A., HOUGHTON, J., LEAPER, D. J. & STAMFORD, I. F. (1978) Tumour growth and response to treatment: Beneficial effect of the prostaglandin synthesis inhibitor flurbiprofen. *Br. J. Pharmacol.*, **63**, 356p.

BENNETT, A., HOUGHTON, J., LEAPER, D. J. & STAMFORD, I. F. (1979) Cancer growth, response to treatment and survival time in mice: Beneficial effect of the prostaglandin synthesis inhibitor flurbiprofen. *Prostaglandins*, **17**, 179.

BENNETT, A., STAMFORD, I. F. & UNGER, W. G. (1973) Prostaglandin $E_2$ and gastric acid secretion in man. *J. Physiol.*, **229**, 349.

BENNETT, A., BERSTOCK, D. A. & CARROLL, M. A. (1981) Enhanced anti-cancer effect by combining cytotoxic drugs with the prostaglandin synthesis inhibitor flurbiprofen. *Br. J. Pharmacol.*, **71**, 208P.

BERSTOCK, D. A., FRANK, G. J., STAMFORD, I. F. & BENNETT, A. (1980) Decrease in aspirin-induced gastric mucosal damage in rats by oral administration of the cytotoxic drugs melphalan and methotrexate. *J. Pharm. Pharmacol.*, **32**, 544.

BERSTOCK, D. A., HOUGHTON, J. & BENNETT, A. (1979) Improved anti-cancer effect by combining cytotoxic drugs with an inhibitor of prostaglandin synthesis. *Cancer Treat. Rev.*, **6** (Suppl.), 69.

FAVALLI, C., GARACI, E., ETHEREDGE, E., SANTORO, M. G. & JAFFE, B. M. (1980) Influence of PGE on the immune response in melanoma-bearing mice. *J. Immunol.*, **125**, 897.

GASIC, G. J., GASIC, T. B., GALANTI, N., JOHNSON, T. & MURPHY, S. (1973) Platelet tumor-cell interactions in mice. The role of platelets in the spread of malignant disease. *Int. J. Cancer*, **11**, 704.

GRYGLEWSKI, R. J., PANCZENKO, B., KORBUT, R., GRODZINSKA, L. & OCETKIEWICZ, A. (1975) Corticosteroids inhibit prostaglandin release from perfused mesenteric blood vessels of rabbit and from perfused lungs of sensitised guinea-pig. *Prostaglandins*, **10**, 343.

HEWITT, H. B., BLAKE, E. R. & WALDER, A. S. (1976) A critique of the evidence for active host defence against cancer, based on personal studies of 27 murine tumours of spontaneous origin. *Br. J. Cancer*, **33**, 241.

HIGGS, G. A., EAKINS, K. E., MUGRIDGE, K. G., MONCADA, S. & VANE, J. R. (1980) The effect of non-steroid anti-inflammatory drugs on leukocyte migration in carrageenin-induced inflammation. *Eur. J. Pharmacol.*, **66**, 81.

HOFER, D., DUBITSKY, A. M., REILLY, P., SANTORO, M. G. & JAFFE, B. M. (1980) The interactions between indomethacin and cytotoxic drugs in mice bearing B-16 melanomas. *Prostaglandins*, **20**, 1033.

LEAPER, D. J., FRENCH, B. T. & BENNETT, A. (1979) Breast cancer and protaglandins: A new approach to treatment. *Br. J. Surg.*, **66**, 683.

LEE, E. & DESU, M. (1972) A computer program for comparing K samples with right-censored data. *Computer Programs Biomed.* **2**, 315.

LYNCH, N. R., CASTES, M., ASTOIN, M. & SALOMON, J. C. (1978) Mechanism of inhibition of tumour growth by aspirin and indomethacin. *Br. J. Cancer*, **38**, 503.

MENNIE, S. A. T., DALLEY, V., DINNEEN, L. C. & COLLIER, H. O. J. (1975) Treatment of radiationinduced gastrointestinal distress with acetylsalicylate. *Lancet*, ii, 942.

NORTHOVER, B. J. (1973) The effect of anti-inflammatory drugs on the binding of calcium to cellular membranes in various human and guinea-pig tissues. *Br. J. Pharmacol.*, **48**, 496.

O'CONNOR, A. D., CLIFFORD, P., DURDEN SMITH, D. J. & 3 others (1977) Synchronous V.B.M. and radiotherapy in the treatment of squamous cell carcinoma of the head and neck. *Clin. Otolaryngol.*, **2**, 347.

PLESCIA, O. J., SMITH, A. H., & GRINWICH, K. (1975) Subversion of immune system by tumor cells and role of prostaglandins. *Proc. Natl Acad. Sci.*, **72**, 1848.

POWLES, T. J., ALEXANDER, P. & MILLAR, J. L. (1978) Enhancement of anti-cancer activity of cytotoxic chemotherapy with protection of normal tissues by inhibition of prostaglandin synthesis. *Biochem. Pharmacol.*, **27**, 1389.

POWLES, T. J. & MILLAR, J. L. (1979) Non-steroidal anti-inflammatory drugs and cytotoxics. *Cancer Treat. Rev.*, **6** (Suppl.), 63.

RITCHIE, G. (1978) The direct inhibition of prostaglandin synthetase of human breast cancer tissue by "Novaldex". *Rev. Endocrine-Related Cancer* (Suppl.), 35.

ROBERT, A. (1979) Cytoprotection by prostaglandins. *Gastroenterology*, **77**, 761.

SCHIFFMAN, F. J., UEHARA, Y., FISHER, J. M. & RABINOVITZ, M. (1978) Potentiation of chlorambucil activity by phenylbutazone. *Cancer Letters*, **4**, 211.

TANNER, N. S. B., STAMFORD, I. F. & BENNETT, A. (1981) Plasma prostaglandins in mucositis due to radiotherapy and chemotherapy. *Br. J. Cancer*, **43**, 767.

TREVISANI, A., FERRETTI, E., CAPUZZO, A. & TOMASI, V. (1980) Elevated levels of prostaglandin $E_2$ in Yoshida hepatoma and the inhibition of tumour growth by non-steroidal anti-inflammatory drugs. *Br. J. Cancer*, **41**, 47.

UNGER, W. G., STAMFORD, I. F. & BENNETT, A. (1971) Extraction of prostaglandins from human blood. *Nature*, **233**, 336.